# Intranasal Delivery of Recombinant S100A8 Protein Delays Lung Cancer Growth by Remodeling the Lung Immune Microenvironment

**DOI:** 10.3389/fimmu.2022.826391

**Published:** 2022-05-17

**Authors:** Sze Wing Wong, Joshua McCarroll, Kenneth Hsu, Carolyn L. Geczy, Nicodemus Tedla

**Affiliations:** ^1^ School of Medical Sciences and the Kirby Institute, Faculty of Medicine, UNSW Sydney, Sydney, NSW, Australia; ^2^ Children’s Cancer Institute, Lowy Cancer Research Centre, UNSW Sydney, Sydney, NSW, Australia; ^3^ Australian Centre for Nanomedicine, UNSW Sydney, Sydney, NSW, Australia; ^4^ School of Women’s and Children’s Health, UNSW Sydney, Sydney, NSW, Australia

**Keywords:** lung cancer, S100A8, myeloid-derived suppressor cells, antioxidants, lung immune microenvironment

## Abstract

Lung cancer is the leading cause of cancer-related death worldwide. Increasing evidence indicates a critical role for chronic inflammation in lung carcinogenesis. S100A8 is a protein with reported pro- and anti-inflammatory functions. It is highly expressed in myeloid-derived suppressor cells (MDSC) that accumulate in the tumor microenvironment and abrogate effective anti-cancer immune responses. Mechanisms of MDSC-mediated immunosuppression include production of reactive oxygen species and nitric oxide, and depletion of L-arginine required for T cell function. Although S100A8 is expressed in MDSC, its role in the lung tumor microenvironment is largely unknown. To address this, mouse recombinant S100A8 was repeatedly administered intranasally to mice bearing orthotopic lung cancers. S100A8 treatment prolonged survival from 19 days to 28 days (p < 0.001). At midpoint of survival, whole lungs and bronchoalveolar lavage fluid (BALF) were collected and relevant genes/proteins measured. We found that S100A8 significantly lowered expression of cytokine genes and proteins that promote expansion and activation of MDSC in lungs and BALF from cancer-bearing mice. Moreover, S100A8 enhanced activities of antioxidant enzymes and suppressed production of nitrite to create a lung microenvironment conducive to cytotoxic lymphocyte expansion and function. In support of this, we found decreased MDSC numbers, and increased numbers of CD4^+^ T cells and natural killer T (NK-T) cells in lungs from cancer-bearing mice treated with S100A8. *Ex-vivo* treatment of splenocytes with S100A8 protein activated NK cells. Our results indicate that treatment with S100A8 may favourably modify the lung microenvironment to promote an effective immune response in lungs, thereby representing a new strategy that could complement current immunotherapies in lung cancer.

## Introduction

The host response to cancer can induce a powerful immune response that influences clinical outcomes (1, 2). This is illustrated by the recent expansion of the use of immunotherapies such as immune checkpoint inhibitors (CTLA-4, PD-1 and PD-L1), which harness the host immune system to generate powerful anti-cancer effects (3). Indeed, several cancers, including non-small cell lung cancer (NSCLC), have witnessed significant improvements in patient survival with the implementation of immune checkpoint inhibitors (3). However, despite their promise, some patients do not respond to immunotherapies, or develop resistance and relapse (4). A greater understanding of resistance mechanisms and new therapeutic strategies with the potential to synergize with immunotherapies or enhance their effectiveness by priming the cancer microenvironment is required.

A neoplastic microenvironment characterized by infiltrating myeloid-derived suppressor cells (MDSC) (5) and high oxidative stress is generally immunosuppressive (6, 7) and negatively contributes to the pathogenesis of lung cancer (8, 9). MDSC accumulation in cancers also suppresses CD8^+^ T cell activation and hinders the efficacy of PD-1 and CTLA-4 checkpoint inhibitors (10–12). Emerging studies designed to identify key mediators which can alleviate immunosuppression and modify an unfavourable microenvironment show promise (13). For example, in murine models of lung cancer, direct inhalation of immunostimulatory CpG-ODN motifs, which activate Toll-like Receptor 9 (TLR9) signaling, stimulated anti-cancer activity by modifying the tumor microenvironment in favour of increased recruitment and activation of T cells (14). Moreover, the synergy between inhaled CpG-ODN and programmed cell death-1 (PD-1) inhibitors increased survival (14). In another study, CpG-ODN was combined with Poly I:C (TLR3 agonist) and an anti-MDSC antibody (RB6-8C5) and delivered as a cocktail locally to lungs of mice suffering from metastatic melanoma to the lungs (15). This treatment strategy favourably modified the lung cancer immune microenvironment by inhibiting expression of key immunosuppressive genes and increasing activity of resident NK cells.

Numerous studies report clinical associations between elevated expression of members of the S100 family of calcium-binding proteins and human malignancies (16), but their functional significance remains limited and conflicting. S100A8 and S100A9 are constitutively expressed in neutrophils (17), MDSC (18) and other cell types that promote progression of cancer (19). Some cancer cells, such as anaplastic thyroid cancer cells, also express high levels of S100A8 and S100A9 (20). Stable knockdown of S100A8 using shRNA in these cells reduced local cancer growth and metastasis to the lungs, whereas S100A9 knockdown had no effect (20). Studies using S100A9 knockout mice, or mice implanted with lung cancer cells in which S100A9 gene expression was silenced, reported that S100A9 promotes MDSC accumulation in the lungs (21) and may contribute to increased metastasis to the liver (22). In contrast, the S100A8/A9 complex was reported to have anti-cancer activity by increasing natural killer (NK) cell numbers and activity in pancreatic and colon cancer cells transplanted into mice (23). Together, these conflicting results warrant the need for better understanding o the functional roles of S100A8 and/or A9 in the immunopathology of cancers.

We previously made the unexpected finding that when inhaled directly into the lungs of mice, S100A8 promoted anti-inflammatory responses in the airways (24). This suppressed acute lung injury induced by lipopolysaccharide (LPS) by inhibiting expression of key chemokines and cytokines that influence leukocyte recruitment (24). Furthermore, S100A8 inhalation reduced symptoms of acute asthma in mice (25). Its anti-inflammatory effects include the ability to scavenge oxidants generated in the inflammatory milieu without the need to form S100A8/A9 complexes (24). Given that most lung cancers develop on a background of chronic inflammation (26, 27), we hypothesized that the anti-inflammatory properties of S100A8 may limit early-stage growth of lung cancers by favorably modifying the lung immune microenvironment.

Studies herein showed for the first time that intranasal delivery of S100A8 delayed aggressive lung cancer growth and increased survival in a syngeneic orthotopic lung cancer mouse model. S100A8 positively modified the lung microenvironment by decreasing expression of key genes and proteins in the lungs which are involved in regulating MDSC recruitment and activation. Moreover, protective antioxidant activities were increased whereas deleterious nitric oxide levels were decreased in lungs from S100A8-treated mice, together with reduced MDSC numbers, and increased CD4 T cell and natural killer T (NK-T) cell numbers. *Ex vivo* treatment of splenocytes with S100A8 activated splenic NK cells. Taken together, our results indicate that S100A8 favourably modified the cancer microenvironment in the lungs to promote effective immune responses and may represent a new treatment option that complements current therapeutic strategies in lung cancer.

## Materials and Methods

### Preparation and Purification of Murine Recombinant S100A8 Protein

A Glutagene pGEX2T-vector containing murine S100A8 and glutathione-S-transferase fusion protein was transformed into *E. coli* BL21QS cells; recombinant murine S100A8 protein was produced and cleaved by thrombin as described (28). S100A8 protein was purified by reverse-phase high-performance liquid chromatography, where it was first eluted from a C8 column (Vydac Separations Group, USA) with a gradient of trifluoroacetic acid (0.095-0.1%) and acetonitrile (5-99%) solvents, followed by an analytical C4 column (Vydac Separations Group, USA) (**Supplementary Figures 1A, B**). The molecular mass of S100A8 protein (10 kDa) was validated by the Bioanalytical Mass Spectrometry Facility, UNSW Sydney as previously described (29) (**Supplementary Figure 1C**). Purified S100A8 was confirmed to be monomeric by both silver staining and Western blotting (**Supplementary Figures 1D, E**). To minimize endotoxin contamination (< 10 pg for every 10 µg of recombinant S100A8), stringent precautions were implemented to all glassware, buffers and media throughout the study as previously described by our laboratory (29). For intranasal treatments in mice, recombinant murine S100A8 (10 µg) was resuspended in 50 µl Hanks’ Balanced Salt Solution (HBSS).

### Cell Culture

Lewis Lung Carcinoma (LLC) cell line (CRL-1642^™^) was purchased from American Type Culture Collection and authenticated by IDEXX BioResearch (USA) using STR profiling. LLC cells were cultured in DMEM medium supplemented with 10% (v/v) fetal bovine serum and 1% (v/v) L-glutamine (Life Technologies, Australia) at 37°C, 5% CO_2_ for a maximum of 6 months, and routinely verified as mycoplasma free.

### Orthotopic Lung Cancer Mouse Model

Specific-pathogen free female C57Bl/6J mice (6-8 weeks old) from Australian BioResources (Moss Vale, Australia) were maintained in ventilated cages exposed to a 12-hour light or dark cycle and given autoclaved food and water *ad libitum*. Experiments were conducted in accordance with the guidelines of the National Health and Medical Research Council of Australia; ethics were approved by the Animal Care and Ethics Committee of the University of New South Wales, Australia (12/148B and 16/142B). LLC cells (4 x 10^5^/ml) or DPBS (vehicle control; Life Technologies) mixed with chilled growth factor reduced Matrigel™ (BD Biosciences) (1:1 v/v) were orthotopically injected into murine lungs as previously described (30).

### Assessment of Cancer Engraftment Following S100A8 Treatment

*Single treatment:* S100A8 (10 µg) or HBSS (control) was administered intranasally at the same time of orthotopic LLC cell injection into the lungs (Day 0), and whole lungs harvested 20 days later. *Multiple treatments:* S100A8 (10 µg) or HBSS was intranasally administered on Days 0, 3 and 6 post-LLC cell implantation into the lungs, and whole lungs harvested 9 days later. At study endpoints lung cancer areas on hematoxylin and eosin-stained sections were quantified using the CellSens software coupled to an Olympus DP73 microscope at 100X magnification.

### Assessment of Mouse Survival Following S100A8 Treatment

Mice were administered intranasal S100A8 (10 µg) or HBSS on Days 3, 6 and 9 post-LLC cell implantation into the lungs, or every third day. Endpoint criteria included weight loss equal to or greater than 20%, dehydration, lethargy, and labored breathing.

### Assessment of Gene and Protein Expression in the Lung Microenvironment Following S100A8 Treatment

To examine effects of S100A8 inhalation on the lung microenvironment, mice were administered S100A8 (10 µg) or HBSS intranasally on Days 3, 6 and 9 post-LLC cell implantation into the lungs. Cancer-free mice treated with S100A8 or HBSS served as controls. Twenty-four hours after the final treatment (Day 10), lungs were homogenized for gene expression analysis or measurement of antioxidant enzymatic activities, or fixed in 10% neutral-buffered formalin for immunohistochemistry. Bronchoalveolar lavage fluid (BALF), collected by inserting a 19G cannula (Cadence Science, USA) into the trachea and instillation of chilled DPBS (1 ml x 2) into the lungs, was used to quantitate cytokine and nitrite production.

### RT-qPCR Gene Expression Array

Total RNA (1.5 µg) from lungs (≥ 4 mice per treatment group) was reversed-transcribed into cDNA using the SuperScript VILO cDNA synthesis kit (Life Technologies, Australia). Effects of S100A8 treatment on the lung microenvironment were assessed using a panel of 98 genes that affect immune regulation, redox, cancer growth, metastasis, hypoxia and angiogenesis (**Supplementary Table 1**). Primers for each gene were validated as described previously by our laboratory (24). PCR amplification with a cut off threshold of 38 cycles was performed using the QuantStudio RT-qPCR machine (Roche). Gene expression was normalized against two housekeeping genes, β-actin and βeta-2 microglobulin (β2M), and expressed as means ± SEM for each group. Fold changes with respect to control (HBSS + vehicle; no cancer) were calculated using the web-based software package and Excel-based analysis tool, as previously described (24). Data files were uploaded in the Gene Expression Omnibus (GEO accession number, GSE190817).

### Enzyme-Linked Immunosorbent Assay

Cytokine (IL-1β, IL-4, IL-6, IL-12β, IFN-γ) levels in BALF were measured using enzyme-linked immunosorbent assays (ELISA) according to the manufacturer’s instructions (IL-12β ELISA kit from BioLegend, USA; others from R&D Systems, USA).

### Nitric Oxide Production Assay

Nitric oxide production, in terms of concentration of nitrite (μM), was measured in BALF using the Griess reagent (Sigma); A_540nm_ was measured using a SpectraMax M3 plate reader.

### Antioxidant Enzyme Activity Assays

Lung homogenates (50 mg lungs/ml buffer) were prepared in chilled lysis buffer (50 mM PBS, 1 mM EDTA, 10 μM butylated hydroxytoluene) containing complete proteinase inhibitor cocktail mixture (Roche) using a rotating piston (500 RPM). Absorbance correlated to the activities of superoxide dismutase (SOD) (in terms of purpurogallin formation at 405 nm), thioredoxin reductase (TXN-R) (in terms of 5-thio-2-nitrobenzoic acid formation at 412 nm) and peroxiredoxin (PRDX) (in terms of the inverse of iron (III)-xylenol orange complex formation at 560 nm) in total lung lysates was determined by the SpectraMax M3 plate reader with a path length of 1 cm. Specific enzyme activity of SOD, TXN-R (nmol/min/mg of protein) and PRDX (nmol^-1^/min/mg of protein) were calculated using molar extinction coefficients of 2.47 M^-1^ cm^-1^ (31), 13.6 mM^-1^ cm^-1^ (32) and 4.52 x 10^4^ M^-1^ cm^-1^ (33), respectively.

### Immunohistochemistry

Formalin-fixed paraffin-embedded lung sections were stained with in-house generated rabbit anti-S100A8 and S100A9 IgG antibodies (5 mg/ml) as previously described (34), followed by biotinylated anti-rabbit IgG (1:500 v/v) (Santa Cruz, SC-2027). S100A8^+^ and S100A9^+^ myeloid cells in lungs were manually counted over 10 random fields of view of 0.1 mm^2^ using the Olympus DP73 microscope and averaged. Representative images were photographed using the CellSens software coupled to the microscope.

### Preparation of Single Cell Suspensions From Lungs and Spleen

To analyze changes in MDSC and/or lymphocyte populations harvested on Day 10, single cell suspensions were prepared from lungs and spleen. Lungs were digested in 0.7 mg/ml collagenase A, 30 µg/ml bovine pancreatic DNaseI and 35 U/ml hyaluronidase at 37°C for 1 hour, then mechanically disaggregated and ground using a 40 μm strainer (BD Biosciences). To obtain single cell suspensions containing the lymphocyte/monocyte fraction, Percoll centrifugation (1600 RPM, 25 min, break off) was performed to collect the interface between the 60% and 30% gradients as described (35). Spleens were mechanically disaggregated and ground using a 40 µm strainer. Red blood cells in all suspensions were lyzed using ammonium chloride. Single cell suspensions (10^6^ cells) were prepared in 50 µl 0.1% BSA/PBS for flow cytometry analysis.

### Analysis of MDSC and Lymphocyte Populations

To analyze MDSC populations, single cell suspensions from lungs and spleen were stained with APC anti-CD11b (BD Biosciences, #553312), PE-Cy™7 anti-Gr-1 (BD Biosciences, #552985), FITC anti-CD14 (BD Biosciences, #553739) and PE anti-F4/80 antibodies (Thermo Fisher Scientific, #12-4801-82), or IgG controls. Although CD14 is a marker for human monocytic MDSC (36–38), the antibody used in this study also detected murine monocytes and was used to complement F4/80 reactivity to characterize M-MDSC. Lymphocyte populations in lungs and spleen were detected using FITC anti-CD3 (BD Biosciences, #555274), PE anti-NK 1.1 (BD Biosceinces, #557391), PerCP anti-CD4 (BD Biosciences, #553052) and APC anti-CD8 (BD Biosciences, #553035) antibodies, or IgG controls. Multicolor flow cytometry was performed using a BD FACSCalibur™ (BD Biosciences), and data analyzed using the FlowJo software (Tree Star Inc, USA). Gating on the Gr-1^+^ population identified the CD11b^+^/Gr-1^+^ MDSC population (**Supplementary Figure 2A**). Further analysis of this population characterized neutrophilic MDSC (PMN-MDSC; CD14^-^/F4/80^-^) and monocytic MDSC (M-MDSC; CD14^+^ and/or F4/80^+^) (**Supplementary Figure 2A**). Gating on the CD3^+^ population identified NK-T cells (CD3^+^/NK 1.1^+^), whereas gating on the CD3^-^ identified NK cells (CD3^-^/NK 1.1^+^) (**Supplementary Figure 2B**). Further analysis of the CD3^+^/NK 1.1^-^ gate indicated a population of CD4^+^ T cells, CD8^+^ T cells and double-negative T (DNT) cells (CD4^-^/CD8^-^) (**Supplementary Figure 2B**).

### Analysis of NK Cell Activity *Ex-Vivo*

Primary splenocytes from specific-pathogen free female C57BL/6J mice (6 weeks old) were isolated and cultured in RPMI media supplemented with 10% (v/v) fetal bovine serum, 1% (v/v) MEM sodium pyruvate, 1% (v/v) MEM non-essential amino acid solution (all from Invitrogen), 2 mM L-glutamine, 100 U/mL penicillin and 100 μg/mL streptomycin (Sigma-Aldrich, St Louis, MO). Splenocytes (10^6^ cells) were treated with S100A8 (10 µg/ml) for 24 and 48 hours. Cells treated with media served as controls. Subsequently, splenocytes from each group were stained with FITC anti-CD3 (BD Biosciences, #555274), PE anti-NK1.1 (BD Biosciences, #557391), PerCP-eFluor 710 anti-granzyme B (Thermo Fisher Scientific, #46-8898-82) and APC anti-granzyme A antibodies (Thermo Fisher Scientific, #17-5831-82), or isotype- and fluorochrome-matched controls. To determine percentages of granzyme A and granzyme B in NK cells (CD3^-^/NK1.1^+^), flow cytometry was performed using a BD FACSCalibur machine (BD Biosciences), and data analyzed using the FlowJo software (Tree Star Inc) (**Supplementary Figure 2C**). The cutoff quadrants for positive cells were set using high stringency setting based on < 4% non-specific staining on cells incubated with isotype- and fluorochrome-matched controls (**Supplementary Figure 3**).

### Statistical Analysis

Data analyzed were expressed as means ± standard error of the mean (SEM). Mantel-Cox test compared mean survival of mice with orthotopic lung cancers treated with S100A8 or HBSS (control). For all other experiments, Student’s t-test compared changes in means between two treatment groups, and ANOVA in conjunction with Holm–Šídák’s multiple comparison test compared changes in means for more than two treatment groups. P values < 0.05 were considered statistically significant. GraphPad Prism 9.1.0 software was used for data analysis and plotting of graphs.

## Results

### Intranasal S100A8 Delayed Engraftment of Orthotopic Mouse Lung Cancers and Prolonged Survival

**Figure 1:**
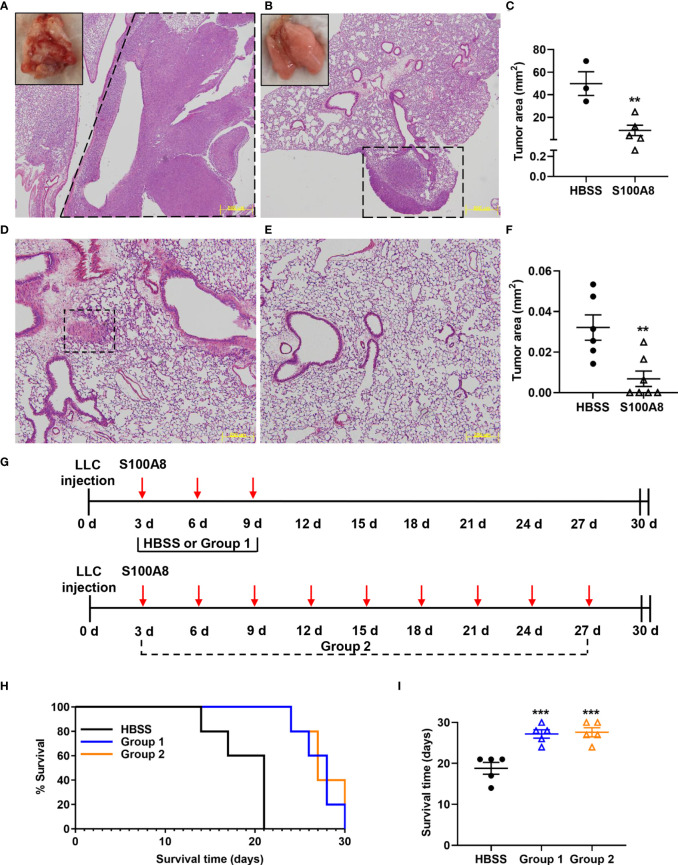
Intranasal S100A8 treatments delayed lung tumor growth and prolonged survival in mice. **(A, B)** Representative Hematoxylin and Eosin (H&E)-stained sections of mouse lungs after a single treatment of Hank’s Balanced Salt Solution (HBSS, control) **(A)** or mouse recombinant S100A8 (10 µg) **(B)**. Lungs were harvested after 20 days, scale bar, 500 μm, dotted line = tumors. Insets show corresponding macroscopic morphology of whole lungs. **(C)** Graph showing mean total tumor area ± SEM (mm^2^) in lungs from mice treated with HBSS or S100A8, n = 3-5 mice per treatment group, **p < 0.01. **(D–F)** Representative H&E-stained sections of mouse lungs after three treatments with HBSS (control) **(D)** or mouse recombinant S100A8 (10 µg) **(E)** on Days 0, 3 and 6 post-LLC cell implantation. Lungs were harvested on Day 9, dotted line = tumors; scale bar, 200 μm. **(F)** Graph showing mean total tumor area ± SEM (mm^2^) in lungs from mice after three treatments with HBSS or S100A8, n = 6-7 mice per treatment group, **p < 0.01. **(G)** Schematic diagrams showing treatment protocols for intranasal S100A8 delivery or HBSS; S100A8 treatments were given on days 3, 6 and 9 post-LLC cell implantation (Group 1) or every third day until endpoint (Group 2). **(H)** Kaplan-Meir curve showing mouse survival for each treatment group (n = 5 mice per treatment group). **(I)** Graph showing the mean survival days ± SEM for mice given S100A8 or HBSS, ***p < 0.001 compared to HBSS.

To determine effects of S100A8 on orthotopic lung cancer engraftment, mice were administered a single intranasal dose of S100A8 or HBSS (control), and whole lungs harvested 20 days post-LLC cell implantation, the time when advanced lung disease was evident. Control mice had extensive lung cancers (HBSS: 49.9 ± 10.5 mm^2^), whereas only small cancer nodules were present in S100A8-treated mice (8.4 ± 4.6 mm^2^) (p < 0.01) (**Figures 1A-C**). When mice bearing LLC cancers were given an additional two intranasal doses on Days 3 and 6 post-LLC cell implantation, striking anti-cancer effects were observed as early as Day 9 (p < 0.01) (**Figures 1D–F**). All control mice developed small cancer nodules throughout the lungs (**Figure 1D**). In marked contrast, S100A8 treatment significantly delayed early lung cancer growth with 4 of 7 (57%) mice having no visible lung cancer nodules (**Figures 1E, F**).

We next examined whether S100A8 treatment increased survival of mice with LLC cancers. Mice were given S100A8 on Days 3, 6 and 9 post-LLC cell implantation (Group 1), or repeated S100A8 treatments every third day for up to 27 days (9 treatments, Group 2); controls received HBSS (**Figure 1G**). Both groups of S100A8-treated mice survived significantly longer (27 ± 1 days, Group 1, p < 0.001 and 28 ± 1 days, Group 2, p < 0.001) compared to controls (19 ± 1 days) (**Figures 1H, I**). Increasing concentrations of S100A8 did not affect proliferation or viability of mouse or human lung cancer cells *in vitro* (**Supplementary Figure 4**), suggesting that S100A8 likely delayed engraftment of lung cancers by alternate mechanisms.

### Inhalation of S100A8 Altered Expression Profiles of Genes Involved in Immune Regulation and Redox in Lungs

**Figure 2:**
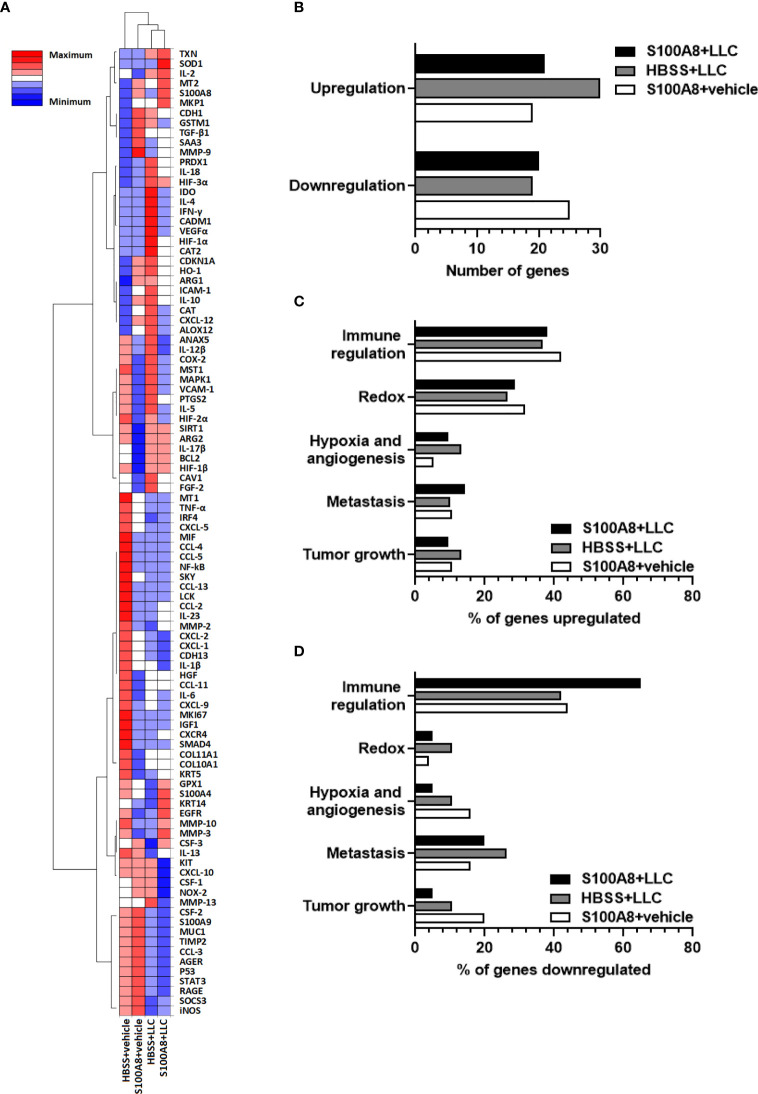
Expression profiles of genes in mouse lungs following intranasal S100A8 treatments. **(A)** Clustergram showing expression of 98 genes in whole lung tissue collected from mice treated intranasally with mouse recombinant S100A8 (10 µg) or HBSS 3, 6 and 9 days post-LLC cell implantation (S100A8 + LLC and HBSS + LLC) and harvested on Day 10. Mice without LLC tumors treated with S100A8 (10 µg) or HBSS served as controls (S100A8 + vehicle and HBSS + vehicle groups). Data from non-supervised hierarchical clustering of the entire data set were combined to display a heatmap with dendrograms indicating co-regulated genes in each treatment group, n = 4-5 mice per treatment group. Increasing intensity of red denotes upregulation by 0.3-fold to 2-fold; maximum denotes changes > 2-fold. Increasing intensity of blue denotes downregulation by 0.3-fold to 2-fold; maximum denotes changes > 2-fold. **(B)** Graph showing total number of genes upregulated or downregulated in each treatment group compared to HBSS + vehicle group. **(C, D)** Graphs showing percentages of genes involved in immune regulation, redox, hypoxia and angiogenesis, metastasis, and tumor growth that were **(C)** upregulated or **(D)** downregulated in each treatment group compared to HBSS + vehicle group.

To investigate potential changes in the lung microenvironment that preceded the delayed lung cancer growth induced by S100A8 treatment, mice with LLC cancers were given three intranasal treatments of S100A8 (Group 1, S100A8 + LLC) as outlined in **Figure 1G**, and whole lungs collected at midpoint of survival (Day 10). Three different control groups were included: 1) HBSS in the absence of LLC cancers (HBSS + vehicle); 2) S100A8 treatment in the absence of LLC cancers (S100A8 + vehicle); and 3) HBSS in the presence of LLC cancers (HBSS + LLC). The relative expression of 98 genes involved in immune regulation, redox, hypoxia, angiogenesis, metastasis, and cancer growth in lungs from the above treatment groups, normalized to HBSS + vehicle, are shown in the clustered heatmap in **Figure 2A**. S100A8 + vehicle upregulated 19 genes and downregulated 25 genes compared to HBSS + vehicle (**Figure 2B** and **Supplementary Table 1**). Lungs from mice with growing LLC cancers (HBSS + LLC) had 30 genes which were upregulated and 19 genes downregulated compared to HBSS + vehicle. Lungs collected from mice treated with S100A8 with growing LLC cancers (S100A8 + LLC) had 21 genes upregulated and 20 genes downregulated compared to HBSS + vehicle (**Figure 2B** and **Supplementary Table 1**). In all treatment groups, most upregulated genes were involved in immune regulation (approximately 40%) and redox (approximately 30%) (**Figure 2C** and **Supplementary Table 1**). Interestingly, most genes downregulated in S100A8-treated lungs were involved in immune regulation; LLC-bearing mice treated with S100A8 (S100A8 + LLC) had the most pronounced effect, with 65% of the downregulated genes involved in immune regulation (**Figure 2D** and **Supplementary Table 1**).

### Inhalation of S100A8 Suppressed Cytokine Production in Lungs and BALF Induced by Orthotopic Mouse Lung Cancers

**Figure 3:**
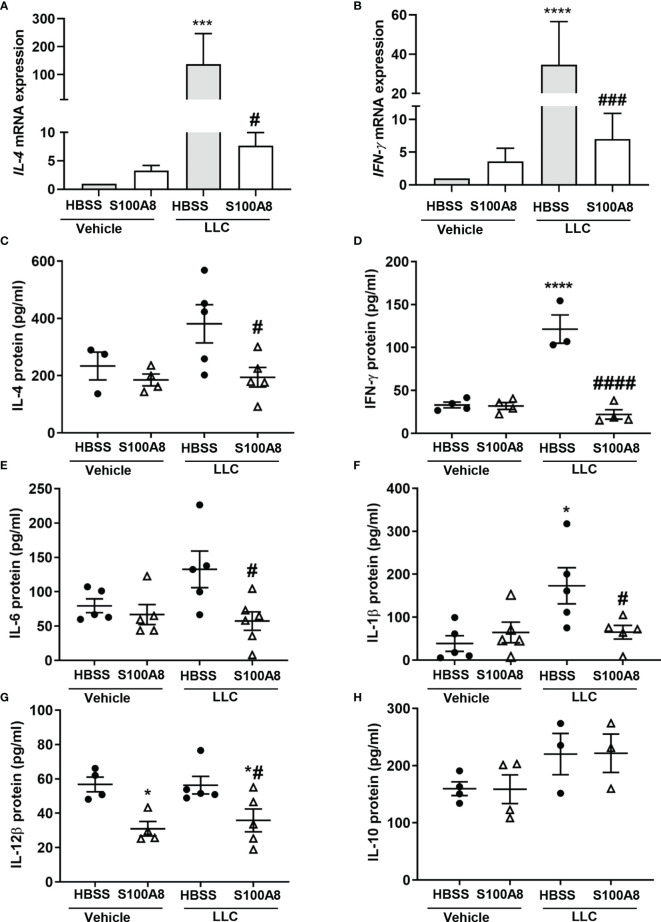
Inhalation of S100A8 suppressed cytokine expression in lungs of mice with orthotopic lung tumors. **(A, B)** Graphs showing mean fold-changes ± SEM of *IL-4* **(A)** and *IFN-γ* **(B)** gene expression in lung tissues from mice treated with recombinant mouse S100A8 on Days 3, 6 and 9 post-LLC cell implantation and collected on day 10. Mice treated with HBSS served as controls, n = 4-5 mice per treatment group. **(C, H)** Graphs showing mean protein concentrations ± SEM (pg/ml) of IL-4 **(C)**, IFN-γ **(D)**, IL-6 **(E)**, IL-1β **(F)**, IL-12β **(G)** and **(H)** IL-10 in bronchoalveolar lavage fluid from mice treated with S100A8 on Days 3, 6 and 9 post-LLC cell implantation and harvested on day 10. Mice treated with HBSS served as controls. *p < 0.05, ***p < 0.001 and ****p < 0.0001 compared to HBSS + vehicle; ^#^p < 0.05, ^###^p < 0.001 and ^####^p < 0.0001 compared to HBSS + LLC.

Of the immunoregulatory genes downregulated by S100A8 in lungs with LLC cancers on Day 10, most were cytokines (**Figure 2A** and **Supplementary Table 1**). *IL-4* and *IFN-γ* mRNA showed the highest upregulation in growing LLC cancers, with increased expression of 137-fold (p < 0.001) and 35-fold (p < 0.0001), respectively, compared to controls (lungs with no cancers) (**Figures 3A, B**). S100A8 treatment significantly reduced expression of both genes by 18-fold (p < 0.05) and 5-fold (p < 0.001), respectively, (**Figures 3A, B**).

Concurrently, S100A8 significantly decreased protein levels of IL-4 (381.0 ± 66.6 pg/mL to 194.3 ± 34.2 pg/mL, p < 0.05) and IFN-γ (121.5 ± 16.5 pg/mL to 22.1 ± 5.5 pg/mL, p < 0.0001) in BALF from mice with LLC cancers to basal levels (**Figures 3C, D**). S100A8 also significantly reduced protein levels of IL-6, IL-1β and IL-12β in BALF from mice with LLC cancers (p < 0.05) (**Figures 3E–G**). IL-12β levels were also decreased in lungs from mice treated with S100A8 +vehicle (p < 0.05) (**Figure 3G**). IL-10 concentrations in BALF from mice with or without LLC cancers were similar (**Figure 3H**), although *IL-10* mRNA expression increased by 80- to 150-fold in response to S100A8 and/or growing LLC cancers (**Supplementary Table 1**). Similarly, there was no significant change in mRNA expression of chemokines or *RAGE* across treatment groups (**Supplementary Table 1**).

### Inhalation of S100A8 Induced Antioxidant Activity and Reduced Nitrite Production in Mice With Orthotopic Lung Cancers

**Figure 4:**
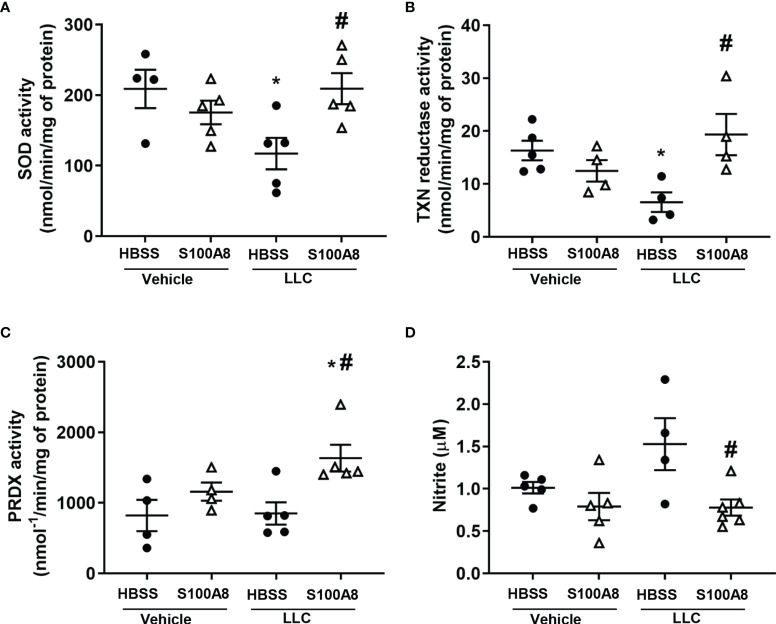
Inhalation of S100A8 induced antioxidant enzyme activity and reduced nitrite production in lungs from mice with orthotopic lung tumors. **(A–C)** Graphs showing enzymatic activities (means ± SEM) of SOD (nmol/min/mg of protein) **(A)**, TXN reductase (nmol/min/mg of protein) **(B)** and peroxiredoxin (PRDX) (nmol^-1^/min/mg of protein) **(C)** in whole lungs collected from mice treated with recombinant S100A8 on Days 3, 6 and 9 post-LLC cell implantation and harvested on Day 10. Mice treated with HBSS served as controls. Mice treated with HBSS served as controls, n = 4-6 mice per treatment group. n = 4-6 mice per treatment group. **(D)** Graph demonstrating mean concentrations of nitrite ± SEM (µM) in bronchoalveolar lavage fluid collected from mice treated with S100A8 on Days 3, 6 and 9 post-LLC cell implantation and harvested on Day 10. Mice treated with HBSS served as controls, n = 4-6 mice per treatment group. *p < 0.05 compared to HBSS + vehicle; ^#^p < 0.05 compared to HBSS + LLC.

Because we previously showed that S100A8 is an oxidant scavenger (39), we next determined whether S100A8 modulated antioxidant enzymes in mice with LLC cancers. S100A8 markedly induced expression of several redox genes on Day 10, including superoxide dismutase (*SOD1*) (5.8 x 10^5^ fold, p < 0.01) and thioredoxin (*TXN*) (1.5 x 10^5^ fold, p < 0.05) in lungs of mice with LLC cancers (**Figure 2A** and **Supplementary Table 1**). Enzymatic activities of SOD and TXN reductase were significantly lower in lungs with growing LLC cancers compared to controls (p < 0.05) (**Figures 4A, B**). Importantly, S100A8 treatment significantly increased activities of these enzymes in lungs with LLC cancers (SOD: 117.1 ± 22.3 to 209.2 ± 22.0 nmol/min/mg protein, p < 0.05; TXN reductase: 6.6 ± 1.9 to 19.3 ± 3.9 nmol/min/mg protein, p < 0.05) to control levels (**Figures 4A, B**). S100A8 also significantly increased peroxiredoxin (PRDX) activity in lungs with growing LLC cancers (850.4 ± 158.8 to 1633.7 ± 190.7 nmol^-1^/min/mg of protein, p < 0.05), although this was not altered in other treatment groups (**Figures 4C**).

S100A8 treatment abolished the elevated nitrite levels found in BALF from mice with LLC cancers (1.5 ± 0.3 μM to 0.8 ± 0.1 μM, p < 0.05, **Figure 4D**), although *iNOS* mRNA did not significantly change regardless of the treatment (**Supplementary Table 1**). Arginase 1 can modulate NO levels by altering arginine availability, and *Arg1* mRNA increased by ~30-fold in response to S100A8 and/or growing LLC cancers (**Supplementary Table 1**). However, we found no change in expression of arginase 1 protein or arginase activity (data not shown). Together, these results suggest that intranasal S100A8 treatment contributes to the reprogramming of the lung microenvironment to reduce ROS and NO and creates a potentially more favorable microenvironment for supporting anti-cancer lymphocytes.

### S100A8 Inhalation Reduced MDSC Accumulation in Lungs and Spleen of Mice With Orthotopic Lung Cancers

The proinflammatory cytokines, IFN-γ, IL-4, IL-6 and IL-1β, promote MDSC expansion and activation (40) and generate reactive oxygen species (ROS) and nitric oxide (NO), which contribute to immunosuppression and reduced T cell function within the tumor microenvironment (41). Given that S100A8 treatment decreased expression of these cytokines (**Figure 3**), induced antioxidant enzyme activity and reduced NO levels (**Figure 4**), we next assessed myeloid cell accumulation in lungs and spleen following S100A8 treatment.

**Figure 5:**
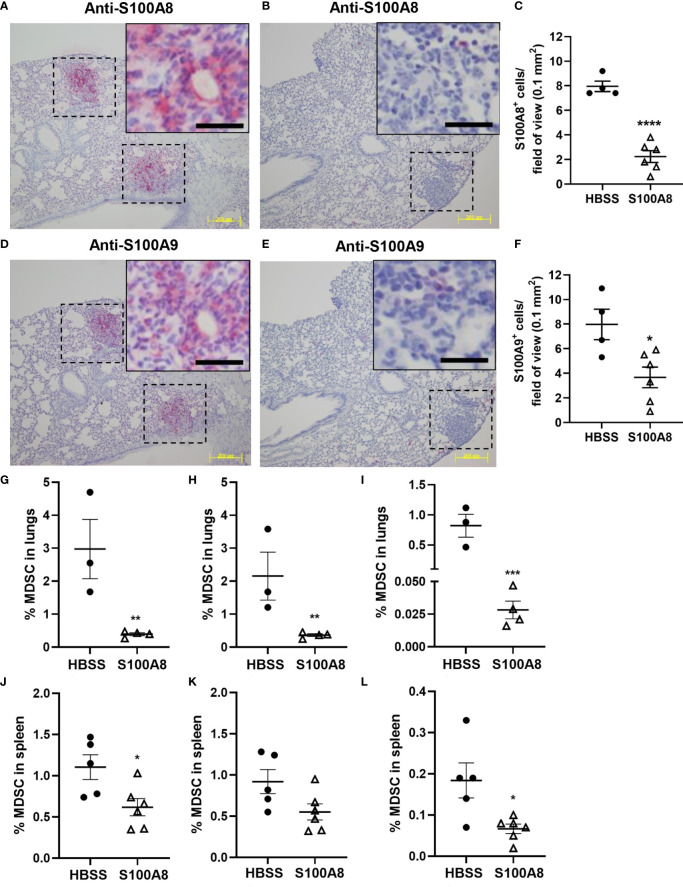
Inhalation of S100A8 suppressed MDSC accumulation in lungs and spleen from tumor-bearing mice. **(A, B)** Representative images showing S100A8 immunoreactivity in lung tumors (dotted lines) following HBSS **(A)** or S100A8 **(B)** treatments on Days 3, 6 and 9 post-LLC cell implantation; scale bars, 200 μm. Insets show magnified regions of tumors; scale bars, 20 μm. Lungs were harvested on Day 10. **(C)** Graph showing mean numbers ± SEM of S100A8^+^ myeloid cells per field of view (0.1 mm^2^), n = 4-6 mice per treatment group, ****p < 0.0001. **(D, E)** Representative images showing S100A9 immunoreactivity in lung tumors (dotted lines) following HBSS **(D)** or S100A8 **(E)** treatments on Days 3, 6 and 9 post-LLC cell implantation and harvested on Day 10; scale bars, 200 μm. Insets show magnified regions of tumors; scale bars, 20 μm. **(F)** Graph showing mean numbers ± SEM of S100A9^+^ myeloid cells per field of view (0.1 mm^2^), n = 4-6 mice per treatment group, *p < 0.05. **(G–L)** Graphs showing mean percentages ± SEM of total MDSC **(G)**, PMN-MDSC **(H)** and M-MDSC **(I)** in lungs and total MDSC **(J)**, PMN-MDSC **(K)** and M-MDSC **(L)** in spleen from tumor-bearing mice treated with HBSS or S100A8 on Days 3, 6 and 9 post-LLC cell implantation and harvested on Day 10, n = 3-6 mice per treatment group, *p < 0.05, **p < 0.01 and ***p < 0.001.

S100A8 and S100A9 are highly expressed in MDSC (18). Immunohistochemical analysis using specific anti-S100A8 and S100A9 antibodies detected S100A8^+^ and S100A9^+^ myeloid cells infiltrating lung cancers (8 cells per field of view) (**Figures 5A–F**), but not in lung tumor cells (**Supplementary Figures 5**, **6**). Importantly, mice treated with S100A8 had significantly less S100A8^+^ and S100A9^+^ myeloid cells in the neoplastic masses (S100A8: 2 cells per field of view, p < 0.0001; S100A9: 4 cells per field of view, p < 0.05) (**Figures 5A–F**), suggesting that S100A8 reduced MDSC accumulation in lungs with cancers.

In line with the above, S100A8 treatment significantly reduced percentages of total MDSC (CD11b^+^/Gr-1^+^), PMN-MDSC (CD11b^+^/Gr-1^+^/CD14^-^/F4/80^-^) and M-MDSC (MDSC that were CD14^+^ and/or F4/80^+^) populations in lungs from mice with LLC cancers (Total MDSC: 2.98 ± 0.90% to 0.39 ± 0.05%, p < 0.01; PMN-MDSC: 2.16 ± 0.73% to 0.36 ± 0.04%, p < 0.01; M-MDSC: 0.82 ± 0.19% to 0.03 ± 0.01%, p < 0.001) (**Figures 5G–I**). Similarly, S100A8 significantly reduced percentages of total and M-MDSC in spleens from mice with LLC cancers (Total MDSC: 1.10 ± 0.15% to 0.62 ± 0.10%, p < 0.05; M-MDSC: 0.18 ± 0.04% to 0.07 ± 0.01%, p < 0.05); PMN-MDSC showed a trend of decrease (**Figures 5J–L**). Percentages of MDSC populations in bone marrow or lymph nodes were not affected by S100A8 in mice with LLC cancers (**Supplementary Table 2**). S100A8 + vehicle did not alter MDSC percentages in any organ (**Supplementary Table 2**).

### Inhalation of S100A8 Increased Lymphocyte Influx Into Lungs of Mice With Orthotopic Lung Cancers and Activated NK Cells

**Figure 6:**
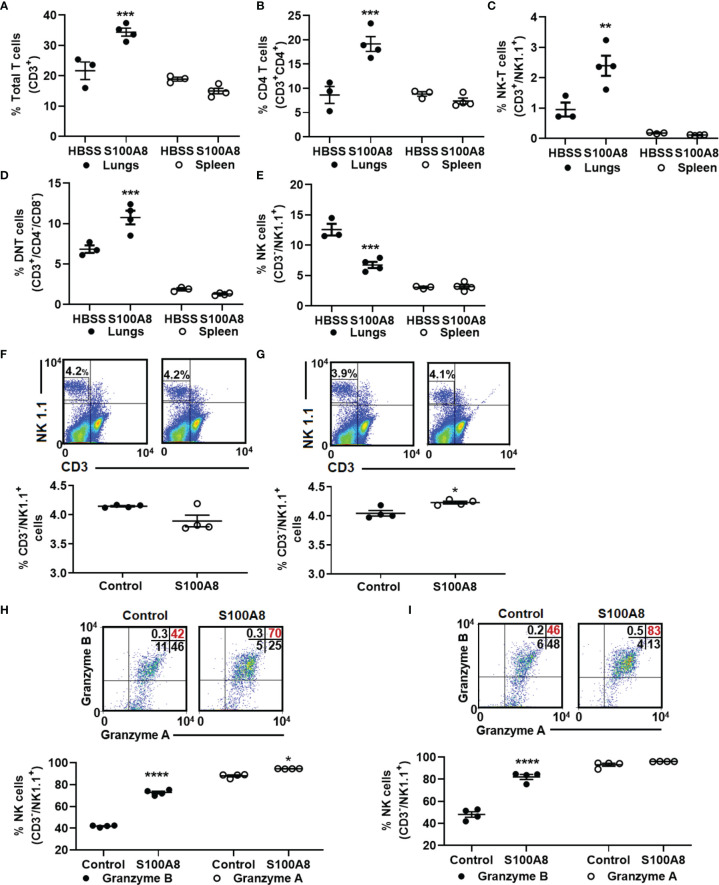
Inhalation of S100A8 increased T cell infiltration into lungs of tumor-bearing mice and activated NK cells *ex vivo*. **(A–E)** Graphs showing mean percentages ± SEM of total T cells (CD3^+^) **(A)**, CD4 T cells (CD3^+^/CD4^+^) **(B)**, NK-T cells (CD3^+^/NK1.1^+^) **(C)**, double-negative T cells (CD3^+^/CD4^-^/CD8^-^) **(D)** and NK cells (CD3^-^/NK1.1^+^) **(E)** in lungs and spleens from tumor-bearing mice treated with recombinant S100A8 or HBSS (controls) on Days 3, 6 and 9 post-LLC cell implantation and harvested on Day 10, n = 3-4 mice per treatment group, **p < 0.01 and ***p < 0.001, **(F, G)** Flow cytometry diagrams and graphs showing mean percentages ± SEM of NK cells isolated from splenocytes from four individual mice **(F)** 24 and **(G)** 48 hours after ex-vivo treatment with recombinant S100A8 (10 µg/ml), *p < 0.05. **(H, I)** Flow cytometry diagrams and graphs showing  mean percentages ± SEM of NK cells expressing granzyme A and granzyme B **(H)** 24 and **(I)** 48 hours after *ex-vivo* treatment with recombinant S100A8 (10 µg/ml), ****p < 0.0001.

Next, we tested whether S100A8 treatment alone altered lymphocyte infiltration and found significantly increased percentage of total T cells (CD3^+^) (21.7 ± 2.9% to 34.4 ± 1.3%, p < 0.001) and their absolute numbers in lungs, but not in spleen, of cancer-bearing mice compared to controls (**Figure 6A** and **Supplementary Table 3**). Among the T cell populations, S100A8 significantly increased the percentages and absolute numbers of CD4 T cells (8.6 ± 1.7% to 19.2 ± 1.5%, p < 0.001, **Figure 6B**, **Supplementary Table 3**), NK-T (CD3^+^/NK 1.1^+^) cells (1.0 ± 0.2% to 2.4 ± 0.3%, p < 0.01, **Figure 6C** and **Supplementary Table 3**) and a population of double-negative T (DNT) cells (6.8 ± 0.5% to 10.8 ± 0.8%, p < 0.001, **Figure 6D** and **Supplementary Table 3**, although changes in spleen were not statistically significant (**Figures 6B-D**). Treatment with S100A8 induced a small but significant increase in percentage (11.1 ± 0.6% to 13.3 ± 0.6%, p < 0.05) and absolute numbers of Tregs (1.1 x 10^5^ to 1.3 x 10^5^ cells) in lungs but not in the spleen (**Supplementary Table 3**). In contrast, S100A8 treatment decreased percentage of NK cells and their absolute numbers in lungs of mice with LLC cancers (CD3^-^/NK1.1^+^) (12.6 ± 1.0% to 6.7 ± 0.5%, p < 0.001); no change was observed in the spleen (**Figure 6E** and **Supplementary Table 3**). S100A8 did not affect the percentage or absolute numbers of CD8 T cells in lungs or spleens from cancer-bearing mice, although it reduced total T cell and DNT cell infiltration in lungs from control mice (p < 0.05) (**Supplementary Table 3**).

Because NK cells infiltrating lung cancers tend to be non-functional and lack cancer-killing activity (42–44), and given that S100A8 treatment reduced percentage of NK cells, but increased percentage of NK-T cells in lungs (**Figures 6C, E**), we next asked whether S100A8 may suppress infiltration of non-functional NK cells and enhance NK cell function. To address this, primary murine splenocytes were isolated and treated *ex-vivo* with S100A8 for up to 48 hours. At 24 hours post-S100A8 treatment, there was no change in NK cell numbers (**Figure 6F**), while at 48 hours post-treatment, S100A8 produced a small but significant increase in NK cell numbers (4.04 ± 0.05% to 4.23 ± 0.02%, p <0.05) (**Figure 6G**). Notably, S100A8 significantly increased percentage of granzyme B^+^ NK cells at 24 (41.8 ± 0.4% to 72.8 ± 1.2%, p < 0.0001) (**Figure 6H**) and 48 hours (48.0 ± 2.5% to 82.1 ± 2.2%, p < 0.0001) post-treatment (**Figure 6I**), indicating increased NK cell activation. S100A8 also slightly increased percentage of granzyme A^+^ NK cells at 24 hours (88.1 ± 0.9% to 94.5 ± 0.1%, p < 0.05) but had no effect at 48 hours (**Figures 6H, I**).

## Discussion

Here we show for the first time that intranasal delivery of S100A8 delayed lung cancer growth in mice. A key finding is that S100A8 positively modified the immune microenvironment by altering expression of key genes and proteins involved in regulating immune cell recruitment and activation as well as redox activity. These changes were associated with profound decreases in numbers of cancer-promoting MDSC and increased infliltration of CD4 and NK-T cells.

Chronic inflammation is increasingly considered a key regulator of lung carcinogenesis [reviewed in (41, 45, 46)]. The lungs are continually exposed to inflammatory stimuli, including smoke, oxidised or non-oxidised inhaled organic and inorganic particles, viruses, and bacteria (47). Normally, epithelial and stromal cells, together with host immune cells, are largely able to deal with these constant insults (47). However, in lung cancer, the microenvironment can become immunosuppressive, promoting prolonged subclinical inflammation leading to sequential metaplastic, dysplastic and eventually neoplastic changes (47). We previously showed that intranasal delivery of recombinant S100A8 produced powerful anti-inflammatory and oxidant-scavenging activities in mouse models of lung inflammation (24, 25). Previous studies support our findings by demonstrating anti-inflammatory properties for S100A8 in different disease states. For example, Sun et al. (48) showed that intraperitoneal administration of recombinant S100A8 protects mice from LPS-induced sepsis by reducing inflammation and oxidative damage in the lungs, kidneys, and liver. Cesaro et al. (49) generated an S100A8 knockout mouse model of collagen-induced arthritis, in which these mice developed a more severe disease than wild-type mice (49). It is therefore plausible that S100A8, by suppressing inflammation, may prevent and/or delay the development of lung cancer. Interestingly, the S100A8/A9 complex is linked to pro-inflammatory activities in numerous cancers and high expression of these proteins is often correlated to increased cancer growth and metastases (50). However, S100A8 can function independently of S100A9 and the S100A8/A9 complex (24, 29). S100A8 was also reported to be pro-inflammatory, by acting as a ligand for Toll-Like Receptor 4 (TLR4) (51, 52) and/or Receptor for Advanced Glycation End Product (RAGE) (53) in mouse models of endotoxin-induced inflammation, possibly suggesting organ- or model-specific functions.

In lung cancer, S100A8 and S100A9 are expressed in both cancer and stromal cells and high expression has been correlated with positive and negative clinical outcomes (54, 55). Studies examining functions of S100A8 in lung cancer , however, are limited; most claiming that its high expression promotes neoplastic growth and metastases (22, 56–58). However, several of these studies used subcutaneous mouse tumor models which are a poor mimic for the lung microenvironment, or focused on manipulating S100A8 expression in cancer cells and examining metastases to the liver or lungs, thereby completely ignoring potential contributions of the complex non-cancerous lung microenvironment. To address this shortcoming, we used an orthotopic syngeneic lung cancer mouse model to investigate the global effects of S100A8 on cancer cells and the lung microenvironment. Unexpectedly, inhalation of S100A8 shortly after implantation of cancer cells into the lungs delayed cancer growth, leading to increased survival. S100A8 can directly affect proliferation and viability of some cancer cell types. Duan et al. (59) showed that recombinant S100A8 promoted proliferation of colorectal carcinoma cells and genetic inhibition of S100A8 expression using shRNA decreased endometrial carcinoma cell proliferation (60). In this study, we showed that the growth and viability of mouse and human lung cancer cells treated with S100A8 over 7 days in culture did not change, indicating that S100A8 may regulate tumor growth or spread of different cancers through different mechanisms. Because S100A8 did not directly alter proliferation of LLC cells, we proposed that its effects might be via modulating the lung microenvironment.

To address this, we determined the expression profiles of 98 relevant genes involved in regulating immune responses, redox activities, and cancer growth in lungs. We observed that treatment with S100A8 decreased expression of key genes that regulate recruitment and activation of immunosuppressive and pro-carcinogenic MDSC. For example, IL-6 mRNA and protein was decreased in lungs and BALF from LLC-bearing lungs after S100A8 inhalation. High IL-6 expression correlates positively with poor survival outcomes in melanoma, head and neck cancers and lung cancers (61). Moreover, high IL-6 in the lung tumor microenvironment associates with increased numbers of immunosuppressive MDSC (61). Similarly, IL-4, IFN-γ and IL-12β expression were also decreased following S100A8 inhalation. These genes are increased in the lung tumor microenvironment of different cancers and activate major signaling pathways that promote MDSC recruitment and activity (61). Importantly, decreased pro-inflammatory gene expression seen in lungs after S100A8 inhalation negatively impacted on MDSC numbers. S100A8 and S100A9 are highly expressed in MDSC and are reported to regulate MDSC activity to promote immunosuppression in cancer (18, 36, 62). We observed S100A8^+^ and S100A9^+^ myeloid cells infiltrating growing lung cancers at midpoint of survival in the orthotopic LLC lung tumour mouse model; PMN-MDSC were the predominant myeloid subset, as reported by others (63, 64). MDSC are implicated in the pathogenesis of lung cancer (47, 65); their depletion from mononuclear cells from human patients with lung cancer was reported to restore CD4 and CD8 T cell functions *ex vivo* (36), and depletion promoted lung cancer rejection, with concomitant increased numbers of CD8 and NK cells in mice (66). Here we made the novel observation that intranasal administration of S100A8 reduced accumulation of PMN-MDSC and M-MDSC in lungs from mice with early-stage lung cancers. These findings contradict an earlier report that used a systemically administered anti-S100A8 antibody to show that S100A8 promoted TLR-4-dependent MDSC recruitment following subcutaneous implantation of LLC cells, thereby establishing a metastatic niche (56–58). However, the specificity of the anti-S100A8 antibody used was not reported, and cross-reactivity with other S100 proteins that are highly structurally homologous (67) is a possibility. Our previous studies show that S100A8 reduced acute lung injury by inducing anti-inflammatory factors such as IL-10 to reduce expression of pro-inflammatory cytokines and chemokines and neutrophil influx, possibly by supressing NF-κB activation via the IkBa/Akt pathway (24). However, mechanisms whereby S100A8 regulates expression of pro-inflammatory cytokines in lungs of mice with lung cancers requires further investigation.

A hypoxic cancer microenvironment is increasingly regarded as a major barrier to the efficacy of chemotherapy and immunotherapies (68). ROS and NO are generated by MDSC (69). In lung cancer, peroxynitrite (70, 71) can promote nitration of T cell receptors to interfere with T cell binding, thereby inhibiting cytotoxic activity (72, 73) and promoting T cell apoptosis (74). N-acetylcysteine, a ROS scavenger, limits the immunosuppressive activity of MDSC in lung cancer mouse models (75), and a SOD mimetic reduced MDSC numbers to enhance CD8^+^ T cell responses in a murine lung cancer model (76). Scharping et al. (77) reported that administration of the mitochondrial complex 1 inhibitor, Metformin, reduced intratumoral hypoxia and increased the efficacy of a PD-1 inhibitor to enhance intratumoral T cell function and cancer clearance in aggressive cancer mouse models. S100A8 is a potent oxygen scavenger (24, 25) and its anti-inflammatory function is partially dependent on its single reactive Cys residue (24) that could have contributed to MDSC suppression shown in this study. Interestingly, here we report that S100A8 increases activities of SOD, TXN and PRDX, enzymes known to scavenge superoxide anions and peroxide oxidants to potentially create a less hypoxic lung cancer microenvironment (78–80) and reduced numbers of cancer-promoting MDSC (68). MDSC also produce NO which can suppress NK cell function (81). Here we show significantly less nitrite levels in bronchoalveolar lavage fluid from mice with lung cancers treated with S100A8, even though *iNOS* mRNA expression was not altered. One explanation could be reduced Arg availability to iNOS caused by increased expression of Arg1, CAT2 or IDO. However, depletion of Arg may not be conducive to effective T cell function, and S100A8 treatment significantly elevated CD3^+^ T cells numbers in lungs of mice with early-stage lung cancers. An alternate possibility is that NO was scavenged by the single Cys_41_ residue in S100A8, shown by us to effectively alter leukocyte adhesion and transmigration (39). Whether S100A8 directly reduced NO production by reducing MDSC numbers to the lung, or shuttled NO to mediators that suppress MDSC activity is unclear, and mechanisms are worthy of future investigation. Together, these observations suggest that S100A8 may promote anti-oxidative effects in the lungs, thereby creating a favourable anti-cancer redox microenvironment. It is noteworthy that expression of several other antioxidant genes such as *HO-1* and *CAT1* were downregulated by S100A8, although we were not able to show any changes in their protein expression or activities.

Immunologically, S100A8 inhalation increased the numbers of CD4^+^ and NK-T cells, but not CD8 T cells, in lungs obtained from mice with orthotopic LLC tumors, which was in marked contrast to its suppressive effects to MDSC numbers. Although these mostly favourable immunological profiles were consistent with the observed delay in lung tumor growth and improved survival, further functional studies are required to examine the effector function of CD4 T cells and to exclude the possibility that they were not Tregs. Moreover, the functions of NK-T cells, NK cells and CD8 T cells as well as their activation status, exhaustion and memory will be examined in future studies to increase our understanding on how S100A8 exerts its anti-inflammatory effects in the lung/immune microenvironment. We also identified a novel double-negative T (DNT) cell population that was elevated in lungs of tumor-bearing mice treated with S100A8 for which the functional significance remains to be elucidated. Somewhat surprisingly, NK cell numbers were reduced in lungs from mice with orthotopic LLC cancers treated with S100A8. Whether the reduction in NK cell numbers is also associated with impaired activation and function remains to be determined. Interestingly, our *ex-vivo* studies showed that S100A8 is a potent activator of NK cells, as indicated by increased expression of granzyme B (82), thus it is plausible that treating tumor-bearing mice with S100A8 may induce activation and function of NK cells without altering their numbers.

Given S100A8 inhalation suppressed but did not abolish lung cancer growth, future studies to determine whether other therapeutic drugs could work in combination with S100A8 are warranted. There is a strong interest in identifying therapeutic strategies that act on the cancer microenvironment to prime an anti-cancer immune response. For example, agonists that activate the cyclic guanosine monophosphate-adenosine monophosphate synthase-stimulation of interferon genes (cGAS-STING) pathway show promise in eliciting a potent anti-cancer immune response in the tumor microenvironment of different cancers including lung cancer (83, 84). Notably, cGAS-STING-agonists act in combination with immune checkpoint targeting drugs (CTLA-4, PD-1, PD-L1) to induce potent cancer eradication (83, 84).

A limitation of this study was the use of a single lung cancer mouse model. Future studies should examine the effects of inhalation of S100A8 on growth of different lung cancer subtypes, or its effect when delivered locally to tumors which rely on an inflammatory and/or redox microenvironment for growth and lung metastasis. In conclusion, our study revealed for the first time that intranasal S100A8 administration to lungs of mice had anti-inflammatory activities, which delayed cancer growth by altering the tumor microenvironment to deplete MDSC and modulate the oxidative balance to encourage accumulation of T cells.

## Data Availability Statement

The datasets presented in this study can be found in online repositories. The names of the repository/repositories and accession number(s) can be found below: GEO, GSE190817 Figshare, DOI: 10.6084/m9.figshare.19365713 after this. 

## Ethics Statement

The animal study was reviewed and approved by Animal Care and Ethics Committee of the University of New South Wales, Australia (12/148B and 16/142B).

## Author Contributions

SW, KH performed experiments. SW, JM, KH, CG and NT have analysed data. JM, CG, and NT designed experiments. SW, JM, CG, and NT wrote the manuscript text. All authors have critically reviewed, read and agreed to the published version of the manuscript.

## Funding

This research was partially funded by the National Health and Medical Research Council of Australia (APP1027189) and APP1144113). JM is in part supported by the Olivia Lambert Foundation.

## Conflict of Interest

The authors declare that the research was conducted in the absence of any commercial or financial relationships that could be construed as a potential conflict of interest.

## Publisher’s Note

All claims expressed in this article are solely those of the authors and do not necessarily represent those of their affiliated organizations, or those of the publisher, the editors and the reviewers. Any product that may be evaluated in this article, or claim that may be made by its manufacturer, is not guaranteed or endorsed by the publisher.

## Supplementary Material

Supplementary Figure 1 Purification of recombinant murine S100A8 protein. **(A)** Representative chromatogram showing elution of recombinant murine S100A8 by reverse-phase high-performance liquid chromatography from a C8 column. Chromatography was performed in a gradient of 5-99% acetonitrile and 0.095%-0.1% trifluoroacetic acid; elution detected at A_214 nm_. The peak corresponding to S100A8 is indicated. **(B)** The S100A8 peak from A was further purified using an analytical C4 column in a gradient of 5-99% acetonitrile (0.095%-0.1% trifluoroacetic acid); elution detected at A_214 nm_. The peak corresponding to S100A8 is indicated. **(C)** Representative mass spectrum of murine S100A8 preparation had a molecular mass of 10307.9653 Da (labelled), concurring with theoretical mass. **(D)** Silver staining of SDS-PAGE gel and **(E)** Western blot of recombinant S100A8 (500 ng) blotted with anti-S100A8 IgG antibody confirmed a single band corresponding to monomeric S100A8.

Supplementary Figure 2 Gating strategies for flow cytometry. **(A)** Representative flow cytometry plots demonstrating the gating strategy for MDSC populations in mouse splenocytes. Gating on the Gr-1^+^ population on the SSC-H verses Gr-1 graph, the CD11b^+^/Gr-1^+^ population was identified as total MDSC. Gating on total MDSC, PMN-MDSC (CD11b^+^/Gr-1^+^/F4/80^-^/CD14^-^) were identified as the predominant cell population; M-MDSC were F4/80^+^ and/or CD14^+^. **(B)** Representative flow cytometry plots demonstrating the gating strategy for lymphocyte populations in mouse splenocytes. Gating on the CD3^+^ population on the SSC-H versus CD3 graph identified NK-T cells (CD3^+^/NK1.1^+^) and NK cells (CD3^-^/NK1.1^+^) cells. Further characterization of the CD3^+^/NK1.1^-^ population identified CD4^+^, CD8^+^ and double-negative (CD4^-^/CD8^-^) T cells. **(C)** Representative flow cytometry plots demonstrating the gating strategy to measure activation of NK cells in mouse splenocytes. Gating on the NK cell population (CD3^-^/NK1.1^+^) identified granzyme A^+^ and granzyme B^+^ (marker of NK cell activation) populations.

Supplementary Figure 3 Staining controls for granzyme A and granzyme B in splenocytes for Figures 6F-6I. **(A, B)** Representative composite plots displaying staining with **(A)** 4-colour isotype and fluorochrome matched antibody controls and **(B)** CD3, NK 1.1, granzyme A and granzyme B for splenocytes treated with media control for 24 hours. **(C, D)** Representative composite plots displaying staining with **(C)** 4-colour isotype- and fluorochrome-matched antibody controls and **(D)** CD3, NK 1.1, granzyme A and granzyme B for splenocytes treated with S100A8 (10 µg/ml) for 24 hours.

Supplementary Figure 4 S100A8 did not affect proliferation of mouse or human lung cancer cells *in vitro*. **(A–C)** Mouse and human lung cancer cells were treated with increasing concentrations of recombinant S100A8 and their growth measured using the CellTiter-Blue^®^ assay over 7 days. Mean fluorescence (A560/590 nm) ± SEM of **(A)** mouse LLC cells treated with 0.2-20 µg/ml of S100A8, and human **(B)** H460 and **(C)** A549 cells treated with 0.1-10 µg/ml of S100A8, n = 3.

Supplementary Figure 5 S100A8 was not detected in LLC tumor cells *in vivo*. **(A–F)** Lungs from mice with orthotopic LLC tumors were harvested after **(A)** 6 days, **(B)** 13 days, **(C)** 18 days, **(D)** 20 days, **(E)** 24 days and **(F)** 30 days. (**D–F** with intranasal S100A8 treatment(s)). Anti-S100A8 reactivity of serial lung sections from LLC-bearing mice shows S100A8 expression in tumor-infiltrating myeloid cells (red cells) but no obvious S100A8 staining in the tumor cells. Sections are representative of at least 3 mice/group; scale bar = 20 μm, dotted lines = tumors.

Supplementary Figure 6 S100A9 was not detected in LLC tumor cells *in vivo*. **(A–F)** Lungs from mice with orthotopic LLC tumors were harvested after **(A)** 6 days, **(B)** 13 days, **(C)** 18 days, **(D)** 20 days, **(E)** 24 days and **(F)** 30 days. (**D–F** with intranasal S100A8 treatment(s)). Anti-S100A9 reactivity of serial lung sections from LLC-bearing mice shows S100A9 expression in tumor-infiltrating myeloid cells (red cells) but no obvious S100A9 staining in the tumor cells. Sections are representative of at least 3 mice/group; scale bar = 20 μm, dotted lines = tumors.

Supplementary Table 1 Expression profiles of 98 genes in lungs of mice treated with or without S100A8 in the presence/absence of orthotopic LLC tumors.

Supplementary Table 2 Total percentage of myeloid-derived suppressor cell (MDSC) populations in lungs, spleen, bone marrow and lymph nodes of mice treated with or without S100A8 in the presence/absence of orthotopic LLC tumors.

Supplementary Table 3 Total percentage and absolute numbers of lymphocyte populations in lungs and spleen of mice treated with or without S100A8 in the presence/absence of orthotopic LLC tumors.
