# Supplementary figures and images for: Intranasal Delivery of Recombinant S100A8 Protein Delays Lung Cancer Growth by Remodeling the Lung Immune Microenvironment
Source: Front Immunol. 2022 May 17;13:826391. doi: 10.3389/fimmu.2022.826391 (PMC9152328; doi:10.3389/fimmu.2022.826391)

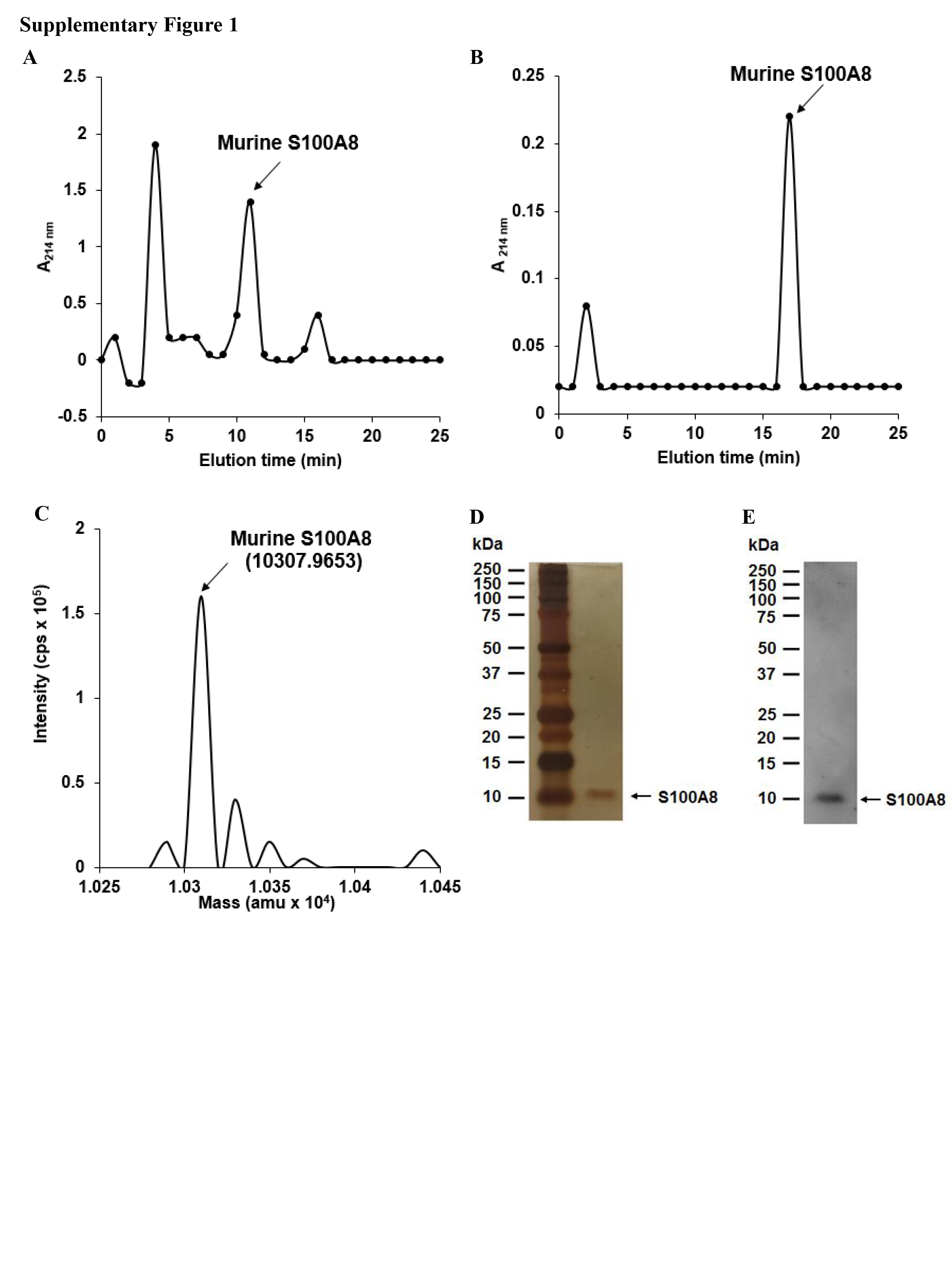

Supplement: Supplementary Figure 1 — Purification of recombinant murine S100A8 protein. (A) Representative chromatogram showing elution of recombinant murine S100A8 by reverse-phase high-performance liquid chromatography from a C8 column. Chromatography was performed in a gradient of 5-99% acetonitrile and 0.095%-0.1% trifluoroacetic acid; elution detected at A214 nm. The peak corresponding to S100A8 is indicated. (B) The S100A8 peak from A was further purified using an analytical C4 column in a gradient of 5-99% acetonitrile (0.095%-0.1% trifluoroacetic acid); elution detected at A214 nm. The peak corresponding to S100A8 is indicated. (C) Representative mass spectrum of murine S100A8 preparation had a molecular mass of 10307.9653 Da (labelled), concurring with theoretical mass. (D) Silver staining of SDS-PAGE gel and (E) Western blot of recombinant S100A8 (500 ng) blotted with anti-S100A8 IgG antibody confirmed a single band corresponding to monomeric S100A8. [file Image_1.tif]

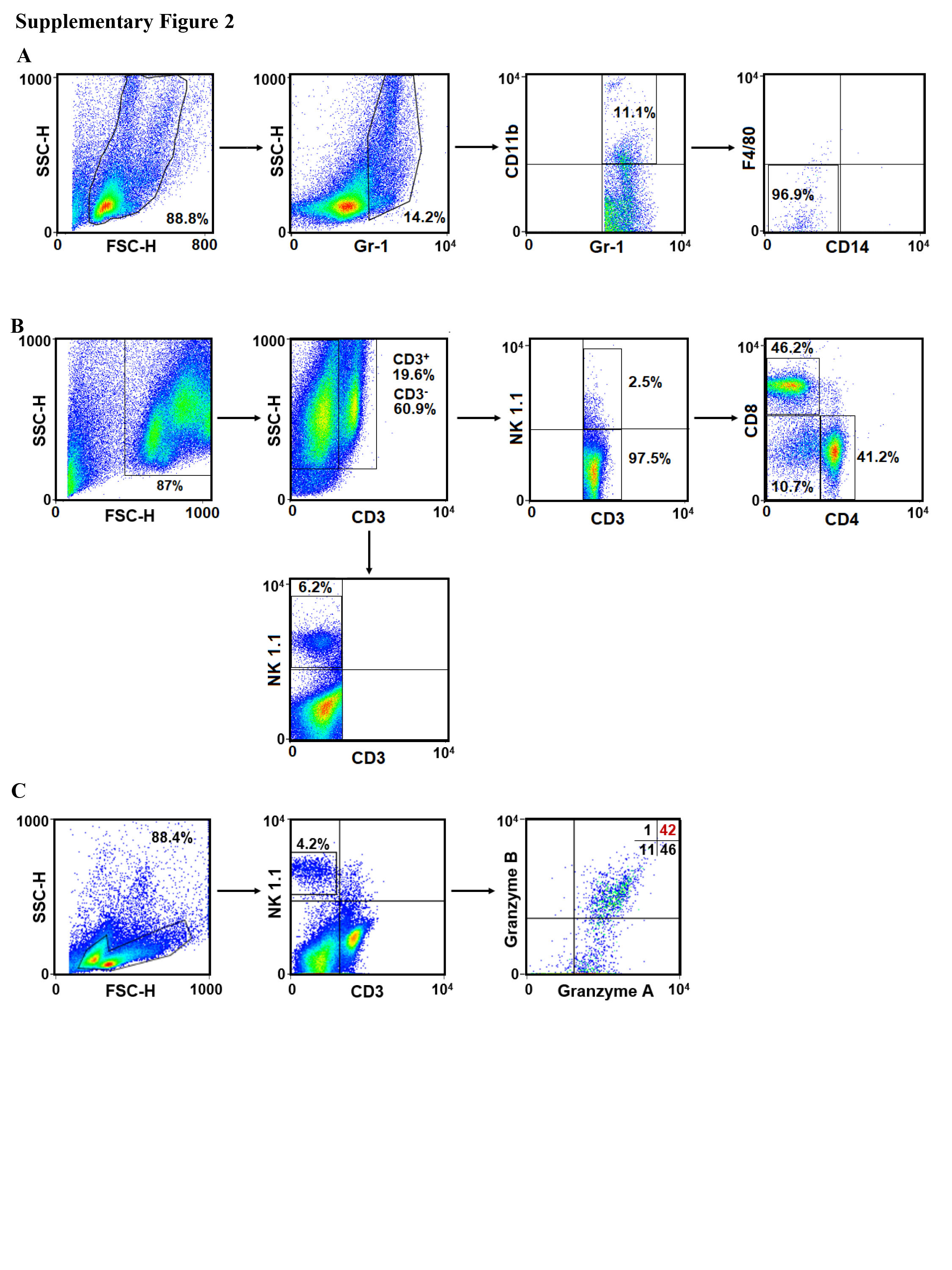

Supplement: Supplementary Figure 2 — Gating strategies for flow cytometry. (A) Representative flow cytometry plots demonstrating the gating strategy for MDSC populations in mouse splenocytes. Gating on the Gr-1+ population on the SSC-H verses Gr-1 graph, the CD11b+/Gr-1+ population was identified as total MDSC. Gating on total MDSC, PMN-MDSC (CD11b+/Gr-1+/F4/80-/CD14-) were identified as the predominant cell population; M-MDSC were F4/80+ and/or CD14+. (B) Representative flow cytometry plots demonstrating the gating strategy for lymphocyte populations in mouse splenocytes. Gating on the CD3+ population on the SSC-H versus CD3 graph identified NK-T cells (CD3+/NK1.1+) and NK cells (CD3-/NK1.1+) cells. Further characterization of the CD3+/NK1.1- population identified CD4+, CD8+ and double-negative (CD4-/CD8-) T cells. (C) Representative flow cytometry plots demonstrating the gating strategy to measure activation of NK cells in mouse splenocytes. Gating on the NK cell population (CD3-/NK1.1+) identified granzyme A+ and granzyme B+ (marker of NK cell activation) populations. [file Image_2.tif]

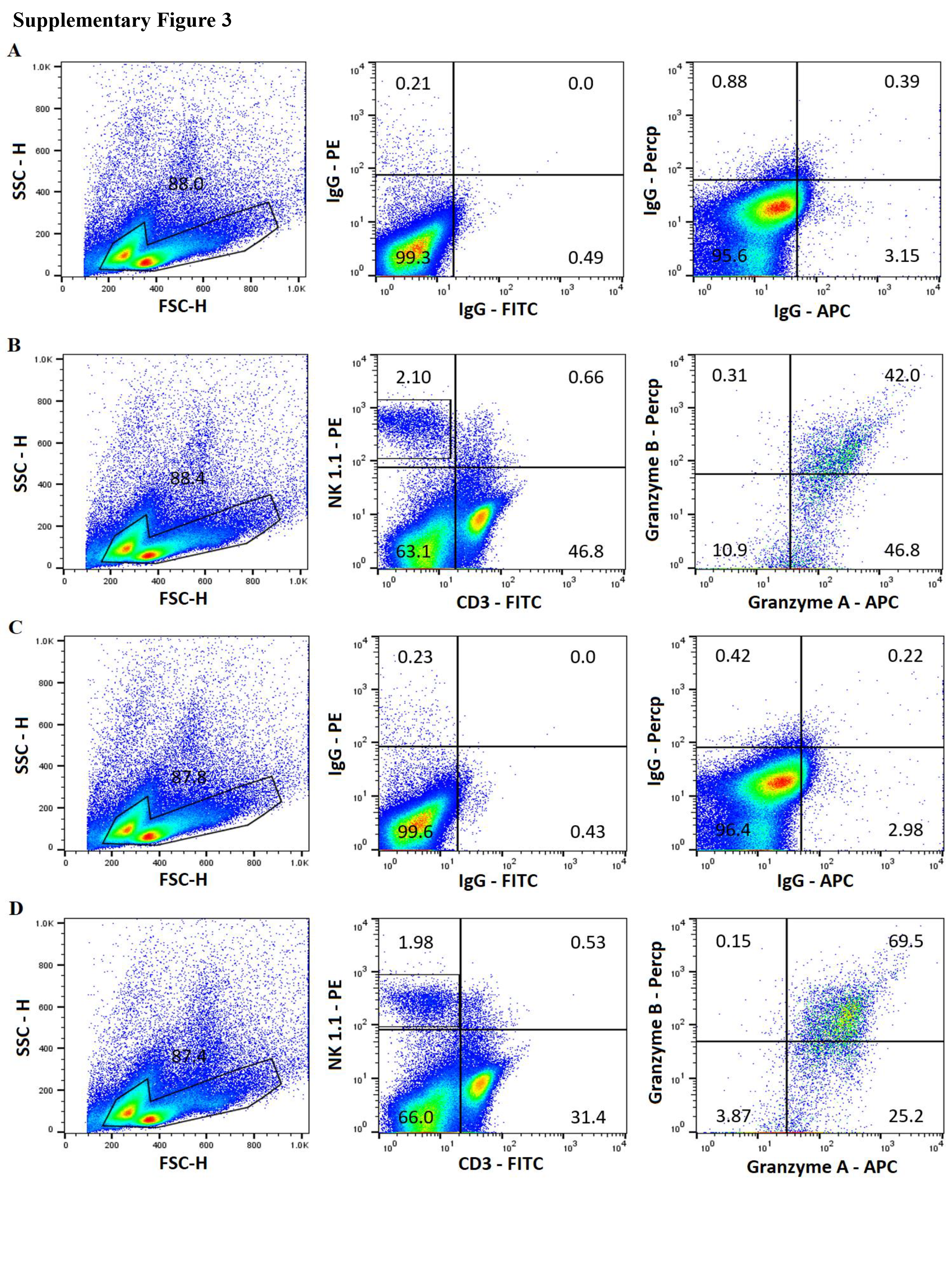

Supplement: Supplementary Figure 3 — Staining controls for granzyme A and granzyme B in splenocytes for Figures 6F-6I. (A, B) Representative composite plots displaying staining with (A) 4-colour isotype and fluorochrome matched antibody controls and (B) CD3, NK 1.1, granzyme A and granzyme B for splenocytes treated with media control for 24 hours. (C, D) Representative composite plots displaying staining with (C) 4-colour isotype- and fluorochrome-matched antibody controls and (D) CD3, NK 1.1, granzyme A and granzyme B for splenocytes treated with S100A8 (10 µg/ml) for 24 hours. [file Image_3.tif]

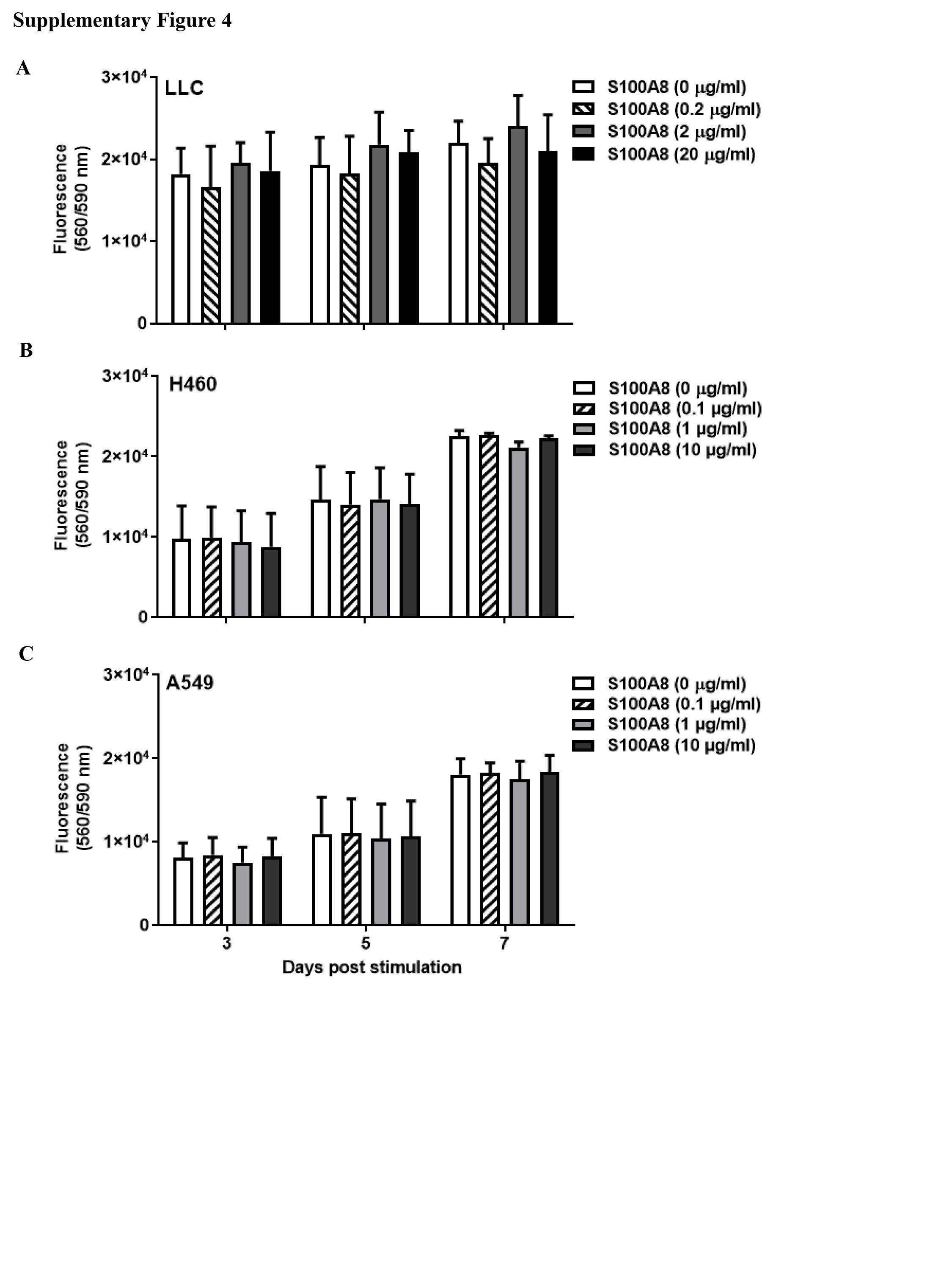

Supplement: Supplementary Figure 4 — S100A8 did not affect proliferation of mouse or human lung cancer cells in vitro. (A–C) Mouse and human lung cancer cells were treated with increasing concentrations of recombinant S100A8 and their growth measured using the CellTiter-Blue® assay over 7 days. Mean fluorescence (A560/590 nm) ± SEM of (A) mouse LLC cells treated with 0.2-20 µg/ml of S100A8, and human (B) H460 and (C) A549 cells treated with 0.1-10 µg/ml of S100A8, n = 3. [file Image_4.tif]

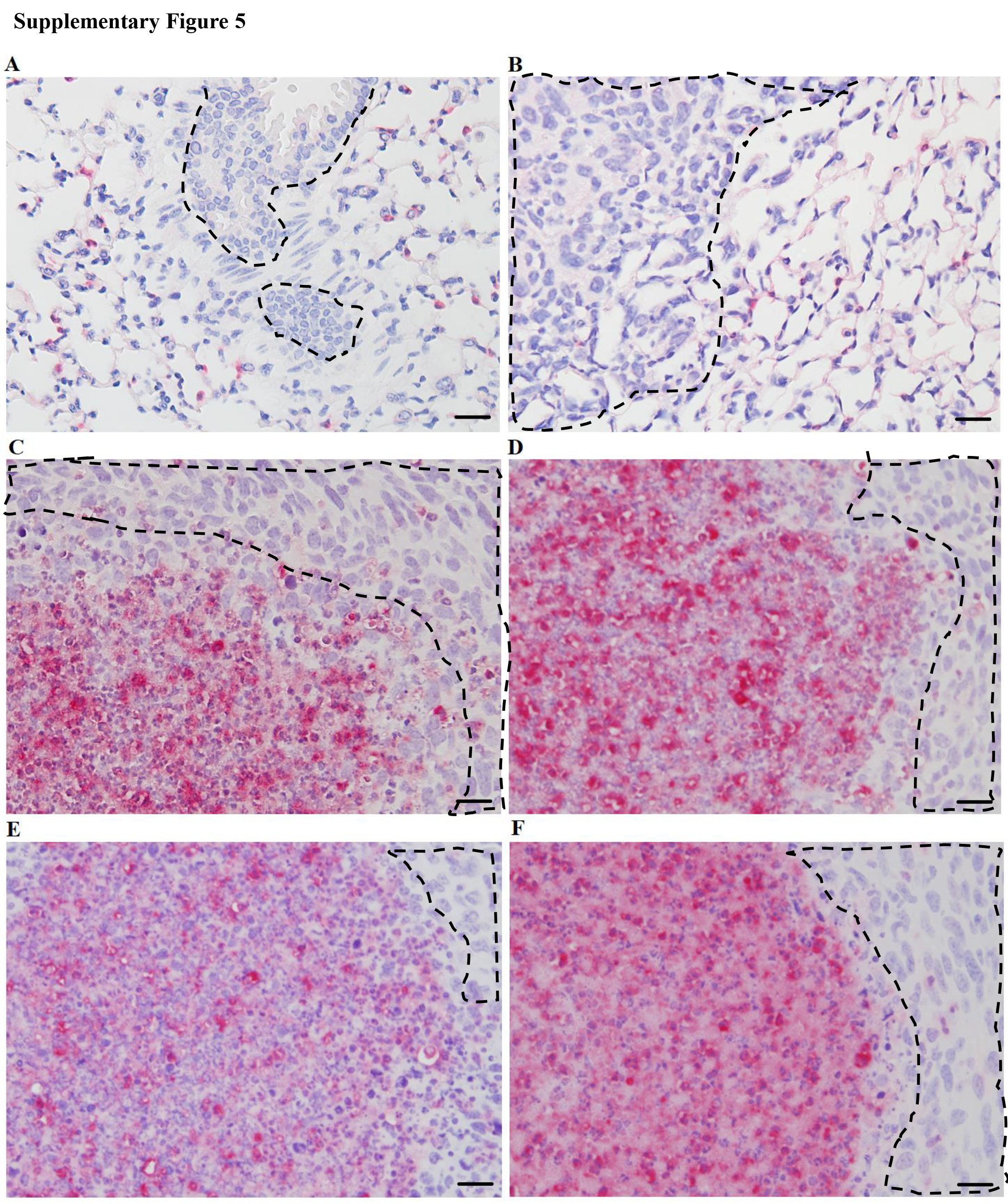

Supplement: Supplementary Figure 5 — S100A8 was not detected in LLC tumor cells in vivo. (A–F) Lungs from mice with orthotopic LLC tumors were harvested after (A) 6 days, (B) 13 days, (C) 18 days, (D) 20 days, (E) 24 days and (F) 30 days. (D–F with intranasal S100A8 treatment(s)). Anti-S100A8 reactivity of serial lung sections from LLC-bearing mice shows S100A8 expression in tumor-infiltrating myeloid cells (red cells) but no obvious S100A8 staining in the tumor cells. Sections are representative of at least 3 mice/group; scale bar = 20 μm, dotted lines = tumors. [file Image_5.tif]

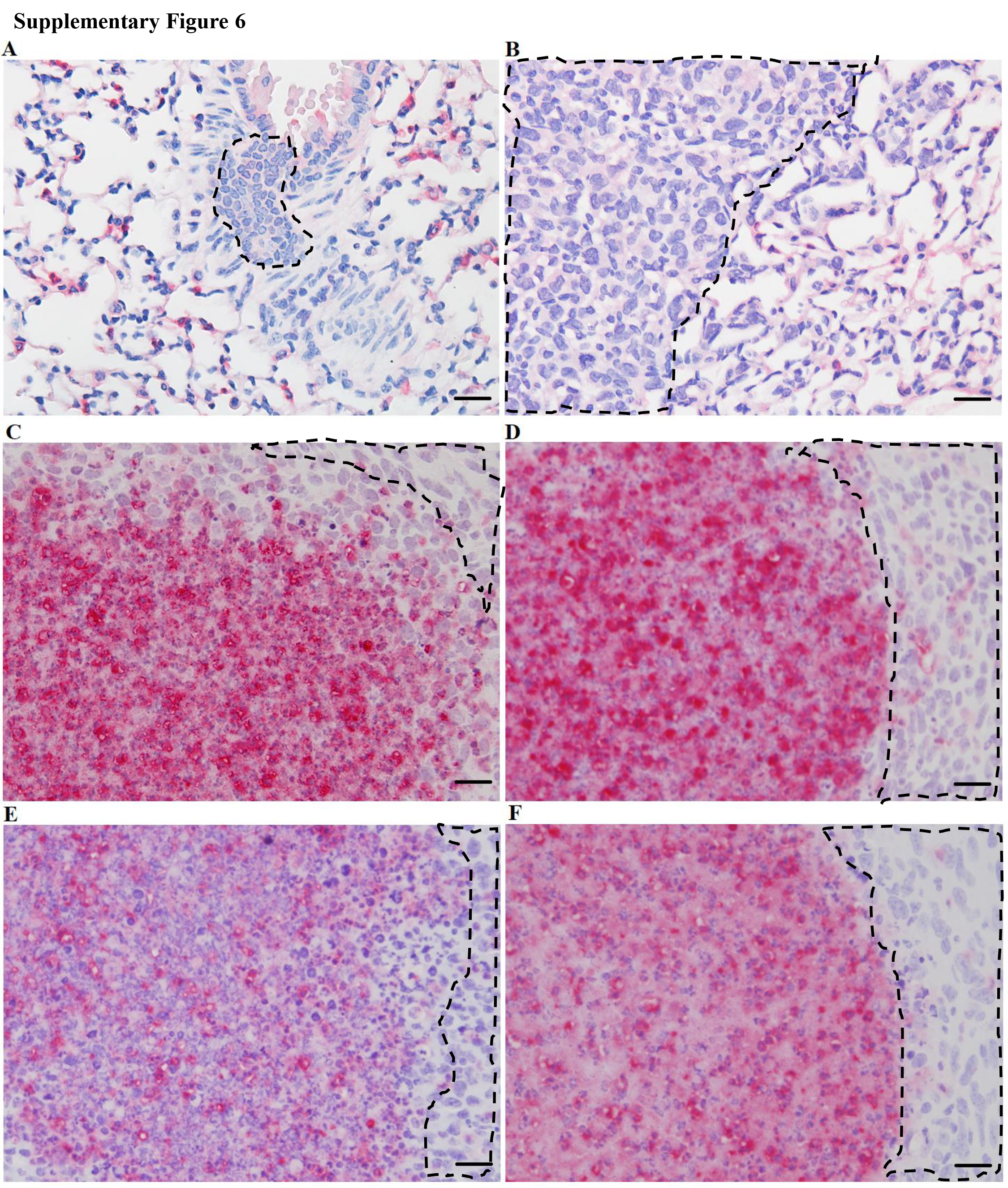

Supplement: Supplementary Figure 6 — S100A9 was not detected in LLC tumor cells in vivo. (A–F) Lungs from mice with orthotopic LLC tumors were harvested after (A) 6 days, (B) 13 days, (C) 18 days, (D) 20 days, (E) 24 days and (F) 30 days. (D–F with intranasal S100A8 treatment(s)). Anti-S100A9 reactivity of serial lung sections from LLC-bearing mice shows S100A9 expression in tumor-infiltrating myeloid cells (red cells) but no obvious S100A9 staining in the tumor cells. Sections are representative of at least 3 mice/group; scale bar = 20 μm, dotted lines = tumors. [file Image_6.tif]
